# Early intervention service intensity and young children’s home participation

**DOI:** 10.1186/s12887-020-02182-x

**Published:** 2020-07-03

**Authors:** M. A. Khetani, B. M. McManus, E. C. Albrecht, V. C. Kaelin, J. K. Dooling-Litfin, E. A. Scully, on behalf of the High Value Early Intervention Research Group

**Affiliations:** 1 Rehabilitation Sciences, University of Illinois at Chicago, Chicago, USA; 2 Occupational Therapy, University of Illinois at Chicago, Chicago, USA; 3 CanChild Centre for Childhood Disability Research, Hamilton, Canada; 4 Health Systems, Management, and Policy, Colorado School of Public Health, Aurora, USA; 5 Invest in Kids, Denver, USA; 6 Rocky Mountain Human Services, Denver, USA

**Keywords:** Young children, Service intensity, Participation, Environment, early intervention

## Abstract

**Background:**

Young children with developmental disabilities and delays spend significant amounts of time at home, show decreased participation in home-based activities, and receive home-based early intervention services to improve participation in activities. Yet, knowledge about the relationship between EI service use and children’s home participation in activities remains poorly understood but needed for program improvement. The purpose of this study was to understand the relationships between EI service use and children’s home participation.

**Methods:**

In a cross-sectional design, data were gathered from caregivers (*N* = 139) who enrolled in a pilot trial of the Young Children’s Participation in Environment Measure (YC-PEM) electronic patient-reported outcome (e-PRO), as implemented within 1 month of their child’s next EI progress evaluation. A series of path analytic models were used to estimate EI service intensity as a predictor of parent-reported young children’s home participation 1) frequency, 2) level of involvement, and 3) desired change, adjusting for family and child social and functional characteristics. Models included caregiver perceptions of home environmental support to test its indirect (i.e., mediation) effects on the relationship between EI service intensity and each of the three home participation dimensions.

**Results:**

All three models fit the data well (comparative fit index = 1.00). EI service intensity was not a significant predictor of participation frequency. However, EI service intensity had a significant direct effect on a child’s participation according to level of involvement and desired change, explaining between 13.3–33.5% of the variance in home participation. Caregiver perceptions of environmental support had a small yet significant indirect effect on the relationship between EI service intensity and level of involvement and desired change; these models explained between 18.5–38.1% of the variance in home participation.

**Conclusions:**

EI service intensity has important links with involvement in and desired change for home-based activities. Caregiver perceptions of environmental support appears to be a factor in the relationship between EI service intensity and home participation. Results warrant longitudinal replication with a control group, which would be possible with the implementation of the YC-PEM e-PRO in a routine EI clinical workflow.

**Trial retrospectively registered:**

NCT03904797.

Early intervention (EI) is a primary source of rehabilitation and developmental services for 2–3% of U.S. infants and young children annually [1]. Significant state variability exists with regard to EI service delivery and EI service use varies as a function of select social and clinical characteristics of the children and families enrolled [2, 3]. There is growing evidence to suggest the importance of EI service use and gains in children’s cognitive and social-emotional competencies from EI entry to exit [4]. Despite EI service delivery variability, another critical aspect of EI care should involve implementing strategies to improve children’s participation in valued activities. There is evidence of the range of relevant everyday activities in which young children typically participate [5], select child and family factors associated with a young child’s participation in activities [6], and mixed evidence of the effect of EI service use (i.e., intensity, duration, and location) on related outcomes like parent and family functioning [7]. However, the relationship between EI service use and gains in children’s participation in activities, a patient-centric outcome of EI care, remains poorly understood.

A key barrier to conducting EI participation-focused outcomes research is the lack of harmonized data collection in routine EI practice to capture the child’s participation as an outcome of interest [8]. While family expertise is essential for evaluating the child’s participation when designing and monitoring EI care, EI providers typically rely on face-to-face, semi-structured, in-home interviews to gather family input about the child’s participation for shared decision-making about how to frame the child’s EI care. Lack of standardized and electronic data capture of caregiver perceptions of children’s participation in valued activities limits large scale program evaluation of the role of EI service delivery on these key outcomes. As EI programs nationally transition to electronic data capture [8, 9], the implementation of electronic participation-focused assessment options may be a scalable strategy that EI programs can use to provide families with options for providing their input when designing and monitoring their child’s EI care. Whereas interview approaches yield rich narrative responses to support work with individual families, data from a standardized, electronic participation-focused assessment can be integrated into collection of core EI data elements (i.e., child characteristics and service use metrics) and aggregated to evaluate the role of EI services on children’s participation within and across programs.

An option for electronic data capture of participation is the Young Children’s Participation and Environment Measure (YC-PEM). The YC-PEM is an evidence-based electronic patient-reported outcome (e-PRO) measure that gives caregivers a valid and reliable way to communicate about their child’s current participation and areas of participation need, while also allowing EI programs to aggregate these data to examine trends in this patient-important outcome over time as a function of EI service use [10–12]. YC-PEM content closely aligns with evidence about those activities in which young children take part in the context of family and community life [5]. There is also preliminary evidence in the YC-PEM e-PRO is a feasible and acceptable option for planning services when it is implemented in partnership with EI staff into routine care with individual families [13] (Kaelin V, Albrecht E, Rigau B, Litfin J, Scully E, Murphy N, McManus B, & Khetani MA, on behalf of the High Value Early Intervention Research Group (conditional accept). Pilot implementation of an electronic patient-reported outcome in an early intervention service context. BMC Med Inform Decis-Mak.) [14].

There is need to examine the value of the YC-PEM e-PRO for EI programs with electronic data capture, to inform its implementation in the longer-term. Towards this end, several studies employing the YC-PEM e-PRO have included young children with disabilities ages 0–5 years [13, 15, 16]. Findings repeatedly suggest environmental support is a significant predictor of participation, accounting for up to 42.5% of model variance. However, its relationship with participation outcomes in the presence of service use remains unclear [13, 15–18]. Caregiver perceptions of environmental support might play a key role in the influence of EI services on home-based participation. Yet, to our knowledge, this has not been fully examined.

To address this knowledge gap, the purpose of this study was to estimate the effect of EI service intensity and home environmental support on children’s participation in valued home activities. The home environment was selected because young children spend significant time and commonly receive EI interventions in this setting. We tested two hypotheses: 1) EI service intensity will be significantly positively associated with home participation; and 2) the relationship between EI intensity and home participation will be explained, in part, (i.e., mediated) by caregiver perceptions of environmental support for home participation. Specifically, greater EI service intensity will be associated with greater perceptions of home environmental support which, in turn, will be related to greater home participation. Study results will provide key insights for EI providers and program directors into family-reported participation difficulties. Results could inform EI program improvement and decision-making about the value of implementing e-PROs to accelerate EI patient-centered outcomes research.

## Methods

### Study sample

This study involves secondary analyses of a subset of data that were collected for a single-arm, non-randomized pilot implementation trial of the YC-PEM e-PRO in EI Colorado (Kaelin V, Albrecht E, Rigau B, Litfin J, Scully E, Murphy N, McManus B, & Khetani MA, on behalf of the High Value Early Intervention Research Group (conditional accept). Pilot implementation of an electronic patient-reported outcome in an early intervention service context. BMC Med Inform Decis-Mak.) [14]. Multi-institutional ethics approval was obtained prior to recruitment and data collection (March 2017–August 2018). Participants were primary caregivers and invited by EI staff to enroll within 1 month prior to the child’s next annual progress evaluation. A caregiver was deemed eligible if he/she was: 1) at least 18 years old; 2) could read, write, and speak English; 3) had internet access; and 4) had a child between 0 and 3 years old who received EI for at least 3 months.

More than half of the enrolled children were 24–35 months old (54.0%). Approximately one in every five families earned less than $50,000 annually. On average, children received EI services for close to 14 months, with 5.82 h of EI services received per month (see Table 1).

**Table 1:** Sample characteristics

Characteristic	*N* = 139 (%)	Mean (SD)	Median (IQR)
Child Sex, Male^a^	71 (51.1)		
Child Age (months)			
12 to 24	64 (46.0)		
over 24	75 (54.0)		
Respondent Type (mother or female guardian)	132 (95.0)		
Family Income^a^			
$0–50,000	29 (20.9)		
$50,001-100,000	32 (23.1)		
$100,001+	73 (52.5)		
Service Duration (months)^a^		13.87 (5.70)	
EI Intensity^a^ (hours per month)		6.56 (3.33)	5.82 [4.67, 8.18]

^a^Missing values

### Measures

Data for this study were both collected online and abstracted from an EI program’s database. Variable selection was informed by conceptual frameworks of participation and health service access [19–21] and align with prior studies examining the impact of child, family, and rehabilitation service use characteristics on young children’s participation [15–18, 22].

#### Home participation and environmental support for participation

Young children’s participation in activities was estimated using the home section of the YC-PEM e-PRO [10, 11]. The YC-PEM e-PRO captures caregiver perspectives of their child’s frequency of attending activities, level of involvement, and satisfaction with valued activities. Caregivers evaluated their child’s participation across 13 types of home activities. For each type of activity, caregivers reported on 1) frequency of attendance (i.e., how often the child attends an activity) (8-point scale, from never [0] to once or more each day [7]; 2) level of involvement (i.e., the child’s level of engagement in the activity) (5-point scale, from not very involved [1] to very involved [5]); and 3) their desire for their child’s participation to change (i.e., their dissatisfaction) [yes, no]. Then, caregivers evaluated the impact of environmental features and resources on home participation (3-point scale, from usually helps/usually yes [3] to usually makes harder/usually no [1]). For this study, mean completion time for the home and community sections of the YC-PEM e-PRO was 21.3 min (range = 12.3–29.9) (Kaelin V, Albrecht E, Rigau B, Litfin J, Scully E, Murphy N, McManus B, & Khetani MA, on behalf of the High Value Early Intervention Research Group (conditional accept). Pilot implementation of an electronic patient-reported outcome in an early intervention service context. BMC Med Inform Decis-Mak.).

Four YC-PEM composite scores were calculated. Home frequency and level of involvement were calculated by averaging responses across all 13 home frequency items and involvement items respectively. For desire of change, a mean percent score was calculated by summing the number of “yes” responses across all 13 desire change items, dividing by the number of items for home participation, and multiplying by 100. To compute a perceived home environmental support summary score, we summed responses across the home environmental items and divided the sum by the maximum possible score, and then multiplied by 100. The YC-PEM e-PRO internal consistency reliability ranged from good to excellent in prior studies [10, 16, 17] and for data obtained in this study (ɑ = .82 for home frequency, ɑ = .75 for home involvement, ɑ = .80 for home desire change, ɑ = .75 for home environmental support).

#### Early intervention service use

Data on EI service use were collected via record abstraction. EI service use intensity was derived from estimates of EI service amount (hours) and EI service duration (months). EI service amount was estimated as the total number of hours of EI services accrued by the time of study enrollment. Service duration was calculated by subtracting the date of EI entry (i.e., the child’s date of EI eligibility evaluation) from the date of study enrollment, as reported in months. EI service intensity (hours per month) was then derived by dividing total EI service amount (hours) by EI service duration (months), to yield an estimate of total hours per month of EI services.

#### Child and family characteristics

Caregivers who confirmed their eligibility to participate in the study were directed to an online demographic questionnaire to report on factors that are known predictors of EI service intensity and participation: 1) predisposing characteristics (e.g., child age); 2) enabling characteristics (e.g., caregiver level of education and annual family income); and 3) service need (e.g., activity competence as indicated by a child’s functional task performance).

For this study, the child’s competency in performing tasks was captured using the Pediatric Evaluation of Disability Inventory - Computer Adapted Test (PEDI-CAT) [23, 24]. The PEDI-CAT affords for caregiver assessment of functional task performance, on a 5-point scale, from unable to easy to do. In this study, a PEDI-CAT normative score for daily activities was selected because most items pertained to self-care tasks that typically occur at home and might therefore contribute to home participation outcomes as previously found.

The PEDI-CAT domains have excellent test-retest reliability and have been shown to be significantly related to participation outcomes [10, 24]. Further, when compared to Child Outcomes Summary (COS) scores, which is a consensus rating that is endorsed in EI as a valid indicator of child’s functioning [3], significant associations between PEDI-CAT daily activities scores and COS mobility scores were found (*r =* .31–.39, *p* < .05). Since routine collection of COS was not mandatory at this EI program throughout the data collection period, there was a high percentage of missing COS data and therefore PEDI-CAT data were included in analyses.

### Data analysis

Descriptive statistics and bivariate correlations were assessed using SPSS (see Table 1). Both caregiver education and family income were considered for inclusion in the analyses based on their known relationship to young children’s participation [13, 25, 26]. Due to concerns with multicollinearity, only family income was selected for inclusion in path analyses. To test the first hypothesis that EI service intensity is positively associated with home participation, we fit a series of path analytic models that examined the direct effects of EI service intensity and caregiver perceptions of environmental support for participation on each domain of home participation: 1) frequency, 2) involvement, and 3) desired change, controlling for child’s age, functional performance, and family income. Path analytic models were computed in Mplus version 8 [27].

To test the second hypothesis that the relationship between EI intensity and each domain of home participation will be explained, in part, (i.e., mediated) by caregiver perceptions of environmental support for participation, when the hypothesis that EI intensity was a significant predictor of children’s participation was verified, we included indirect pathways (i.e., mediation models) using the MODEL INDIRECT command in Mplus, with bias corrected bootstrap resampling (1000 samples), in order to improve the accuracy of the standard error estimates [28].

## Results

Of the 776 caregivers approached to participate, 163 (21.0%) enrolled. Of those who enrolled, 134 (82.2%) had complete information of all variables of interest. Thus, the final analytic sample includes 134 caregivers. This represents 17.3% of total program enrollment.

Mean participation frequency was 4.84 (SD = 1.17) out of 7, and mean level of involvement was 3.83 (SD = 0.64) out of 5. On average, caregivers wanted their child’s participation to change in 27.0% of activities in the home setting.

### Home participation frequency

**Fig. 1:**
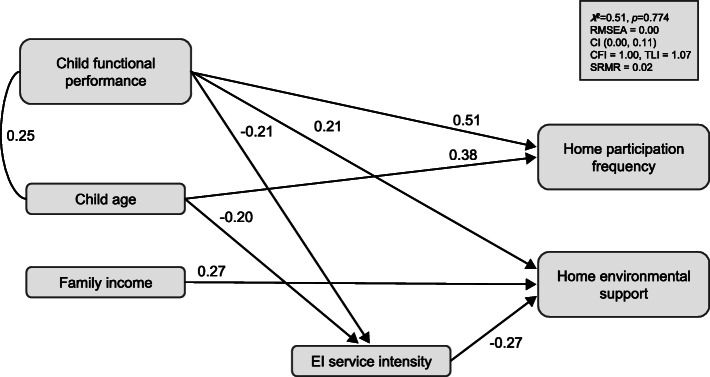
EI intensity, perceived home environmental support, and child and family correlates as predictors of home participation frequency. Significant, completely standardized parameter estimates are shown.

The model provided a good fit to the data across a majority of indices, χ^2^ = 0.54, *p* = 0.774, RMSEA = 0.00, CI (0.00, 0.11), CFI = 1.00, TLI = 1.07, SRMR = 0.02 (see Fig. 1). EI service intensity and home environmental support were not significant predictors of home participation frequency. Child age (*β* = 0.38, *SE* = 0.06) and child functional performance (*β* = 0.51, *SE* = 0.06) of daily tasks predicted home participation frequency, such that older children and children with higher scores in functional performance of daily tasks participated more frequently in home-based activities. Predictors of home environmental support included EI service intensity (*β* = − 0.27, *SE* = 0.09), child functional task performance (*β* = 0.21, *SE* = 0.09), and family income (*β* = 0.27, *SE* = 0.08). Greater EI service intensity was associated with lower levels of perceived home environmental support, whereas greater levels of perceived home environmental support were found in children with higher functional task performance scores and in families reporting higher levels of annual income. Child age (*β* = − 0.20, *SE* = 0.09) and child functional performance (*β* = − 0.21, *SE* = 0.09) predicted EI service intensity, such that older children and children with higher functional performance scores had lower EI service intensity. This model explained 55.5% of the variance in home participation frequency (*p* = 0.000), 20% of the variance in home environmental support (*p* = 0.002), and 10.3% of the variance in EI service intensity (*p* = 0.058).

### Level of home involvement

**Fig. 2:**
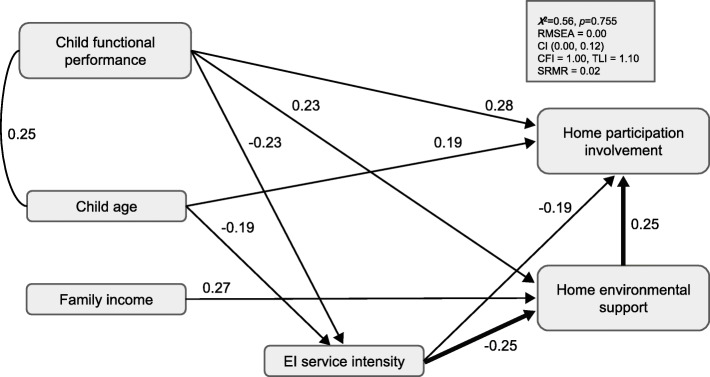
EI intensity, perceived home environmental support, and child and family correlates as predictors of child’s level of involvement in home activities. Significant, completely standardized parameter estimates are shown. Bold arrows depict the estimated indirect effect (i.e., mediation) of EI service intensity on home involvement by way of home environmental support.

The model provided a good fit to the data across the majority of indices, χ^2^ = 0.56, *p* = 0.755, RMSEA = 0.00, CI (0.00, 0.12), CFI = 1.00, TLI = 1.10, SRMR = 0.02 (see Fig. 2). Home environmental support was a significant positive predictor (*β* = 0.25, *SE* = 0.08) of child involvement, such that greater environmental support was related to higher levels of child involvement. EI service intensity was also a significant predictor (*β* = − 0.19, *SE* = 0.09) of home involvement, such that greater service intensity was related to less home environmental support. Additionally, the estimated indirect path from EI service intensity to home participation involvement by way of environmental support (Fig. 2) was statistically significant (*β* = − 0.06, *SE* = 0.03, *p* = 0.028; bias-corrected bootstrapped 95% confidence interval lower limit = − 0.13, upper limit = − 0.02), providing evidence that the role of EI service intensity on home participation involvement may be explained, in part, by caregiver perceptions of environmental support.

In terms of child and family correlates, child functional performance (*β* = 0.28, *SE* = 0.11) and child age (*β* = 0.19, *SE* = 0.07) were significant predictors of home involvement. Specifically, children with higher functional performance scores, and older children demonstrated higher levels of child involvement in home activities. Child functional performance (*β* = 0.23, *SE* = 0.10) and family income (*β* = 0.27, *SE* = 0.09) were significant predictors of home environment support; children with higher functional performance scores and families that reported higher levels of income reported greater perceived home environment support. Child age (*β* = − 0.19, *SE* = 0.09) and child functional performance (*β* = − 0.23, *SE* = 0.10) predicted EI service intensity; older children and children with higher functional performance scores had less EI service intensity. This model accounted for 38.1% of the variance in home involvement (*p* = 0.000), 20.4% of the variance in home environmental support (*p* = 0.005), and 10.6% of the variance in EI service intensity (*p* = 0.088).

### Caregiver desire for home participation change

**Fig. 3:**
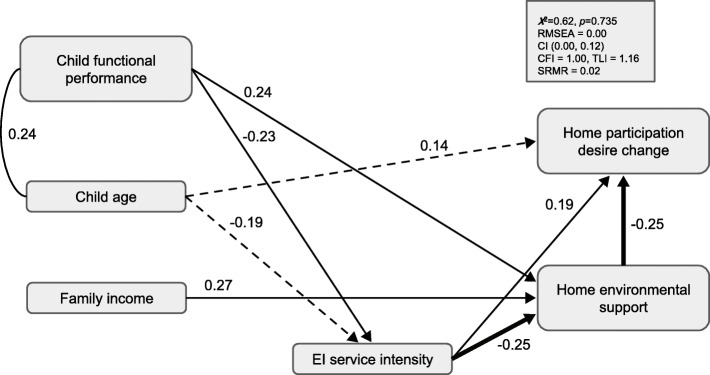
EI intensity, perceived home environmental support, and child and family correlates as predictors of caregiver desire for home participation change. Significant, completely standardized parameter estimates are shown. Bold arrows depict the estimated indirect effect (i.e., mediation) of EI service intensity on desire for change in home participation by way of home environmental support. Dashed lines indicate parameter estimates that showed a trend towards significance (*p* < .10).

The model provided a good fit to the data across a majority of indices, χ^2^ = 0.62, *p* = 0.73, RMSEA = 0.00, CI (0.00, 0.12), CFI = 1.00, TLI = 1.16, SRMR = 0.02 (see Fig. 3). Home environmental support was a significant predictor (*β* = − 0.25, *SE* = 0.10) of desired change, such that greater environmental support was related to less desired change in home participation. EI service intensity was a significant predictor of desired change (*β* = 0.19, *SE* = 0.09), such that greater service intensity was associated with a greater desire for change. Greater service intensity was also significantly related to less home environment support (*β* = − 0.25, *SE* = 0.09). The estimated indirect path from EI service intensity to desired change by way of home environmental support was statistically significant (*β* = 0.06, *SE* = 0.03, *p* = 0.041; bias-corrected bootstrapped 95% confidence interval lower limit = 0.02, upper limit = 0.1), providing evidence that the role of EI service intensity on home participation desired change may be partially explained by parent perceptions of home environmental support.

In terms of child and family correlates, only child age (*β* = 0.14, *SE* = 0.07) showed a trend towards predicting desired change (*p* = 0.063). Similar to the home involvement model, child functional performance (*β* = 0.24, *SE* = 0.10) and family income (*β* = 0.27, *SE* = 0.09) were significant predictors of home environment support; children with higher functional performance scores and families that reported higher levels of income reported a greater desire for change. Child age (*β* = − 0.19, *SE* = 0.09) and functional performance (*β* = − 0.23, *SE* = 0.09) were also associated with EI service intensity. This model accounted for 18.5% of the variance in desire for change (*p* = 0.005), 20.9% of the variance in environmental support (*p* = 0.004), and 10.8% of the variance in EI service intensity (*p* = 0.075).

## Discussion

Young children’s participation in valued activities that take place in supportive environments provides critical early opportunities for promoting positive health and skill development [29]. As the primary source of therapeutic and developmental services for young children with developmental delays and disabilities, EI service delivery has important potential to improve a critical child outcome, home-based participation. Indeed, in an era of accountability, patient-centered outcomes research can help EI programs to demonstrate high value care for relevant outcomes of interest like young children’s participation [30].

This study confirmed and extended evidence for implementing the YC-PEM e-PRO option to advance EI outcomes research in two ways. First, main study findings confirm prior evidence of a significant direct link between EI service intensity and home participation, now across multiple dimensions and while accounting for select child and family characteristics that can likely be extracted via the child’s EI service record. Study results also provide evidence that the role of the home environment should be explored further and contributes to current understanding of how EI service intensity influences home participation involvement and desired change. We discuss both findings in terms of their implications for quality improvement in EI practice.

### EI service intensity and home participation

Our findings suggest that EI service intensity is inversely associated with home participation involvement and positively associated with desired change. However, we did not find evidence that EI service intensity was associated with children’s frequency of attending home activities.

Although we examined three dimensions of home participation, we only found evidence to support the role of EI service use on child’s level of involvement while attending activities and/or caregiver desire for the child’s participation to change, which may be more salient aspects of home participation in the context of EI service receipt. For example, EI services may focus on improving a child’s functional skills to be more involved in activities, as evidenced by prior work showing the significant effect of EI service intensity on gains in cognitive and social-emotional capabilities [3, 31]. We did not have data on EI service type or care quality to know how EI services were focused, but prior studies have found indirect effects of family-centeredness and perceived control over help and services received on a related outcome of parent and family well-being [7]. Therefore, future studies that include data from case progress notes or parent-reported measures of care quality (e.g., family-centered practices) would help to further explain these differential findings across multiple dimensions of the participation outcome [3].

Similar to a prior phase of study, results suggest a negative relationship between EI service use and home participation, despite employing a larger sample of children who had enrolled in EI for approximately twice the duration when compared to the prior study phase [13]. Children receiving more intensive EI services were less involved in activities, and children receiving more intensive EI services had caregivers who expressed greater desire for their child’s participation to change.

One reason for this negative association between EI service use and outcome is that the average duration of EI service use in this study was still not long enough to detect change in participation (e.g., increased levels of involvement, decreased desire for change) to result in a positive association between EI service use and outcome. Participation is an outcome that is known to have slow rates of change [32, 33], so future studies with YC-PEM e-PRO might need to consider follow-up assessment at EI exit, similar to prior work examining the effect of EI service intensity on gains in children’s cognitive and social-emotional functioning.

Alternatively, the significant negative relationships we found between children’s functional performance and EI service intensity across models may suggest a residual selection bias. For example, it is plausible that EI service use is a proxy for developmental need or severity that is not fully captured by including a measure of children’s functional performance. This is consistent with prior work that found more intensive developmental needs was positively associated with service intensity and the duration of services they received [7, 34]. With respect to the current study, children with lower functional performance may express higher clinical need due to condition severity that warrants more intensive EI services and results in lower participation levels and greater participation difficulty. Future studies could employ propensity score estimation to norm-referenced developmental scores extracted from the EI database to create a balance estimate of condition severity to test the association between EI service use and home participation [35, 36].

Since there are no known norms for children’s participation, the finding suggesting the significant role of EI service use on caregiver’s desire for participation change arguably best illustrates the clinical value of including data on participation for EI outcomes research, to detect EI service use as a predictor of parental concerns with their child’s participation. This model accounted for 18.5% of the variance in home participation, which is more than the variance explained in a similar model but involving children with disabilities across a broader age range (0–5 years old) and without accounting for service use [15]. The desire for change dimension may also be responsive to change, given that the average percent desire change is lower in this study as compared to a prior study of children enrolled in EI for a shorter duration [13]. Given its known feasibility, acceptability, and value for highlighting family needs and priorities (Kaelin V, Albrecht E, Rigau B, Litfin J, Scully E, Murphy N, McManus B, & Khetani MA, on behalf of the High Value Early Intervention Research Group (conditional accept). Pilot implementation of an electronic patient-reported outcome in an early intervention service context. BMC Med Inform Decis-Mak.), implementing the YC-PEM e-PRO within EI routine care is a logical next step so that data collected longitudinally can be used to estimate the effect of EI service intensity on gains in participation (i.e., decreased parental desire for change in their child’s participation).

### Home environmental support and home participation

As hypothesized, the role of EI service intensity on home participation involvement and desired change was explained, in small part, by caregiver perceptions of environmental support for the child’s participation in activities. Specifically, more intensive EI services were related to lower perceptions of environmental support which, in turn, was linked to lower levels of involvement and higher percentage of change desired in activities that take place in the home. These findings are consistent with prior studies reporting on the impact of a young child’s environment on his or her home participation [15–18], and may highlight a viable pathway for delivering participation-focused EI care. There are a growing number of evidence-based interventions that emphasize child and family engagement for creating supportive environments for participation, including web-based approaches like PEM-Plus that pull data from the completed YC-PEM e-PRO so that caregivers can design and share preliminary ideas for EI care plans that include participation-focused goals and both child and environmentally-focused strategies for goal attainment [37, 38]. Future studies that include known correlates of home participation, such as parental beliefs and cultural identity that may shape parental expectations for a child’s participation, and/or the child’s developmental status, may help to increase the variance explained by the models showing the indirect effect of home environmental support in explaining the relationship between EI service use and home participation [6]. In contrast to parental beliefs and cultural identity, data on developmental status are routinely collected via the EI service record and may therefore be feasible to expanding knowledge about EI services and outcomes that matter to families.

### Limitations

Study results should be interpreted in light of several limitations. Even though the sample was more diverse than in prior phases of testing, study participants were more educated and affluent than the overall program population. Overall, we had a small sample size and a relative low return rate. This was due to challenges during data collection such as unmet training needs for service coordinators in recruiting participants. More details are described elsewhere [14]. Additionally, we acknowledge limitations of secondary data analysis of EI records for collection of relevant child characteristics and service intensity. We were limited to available data elements and therefore could not include measures of the child’s developmental scores, the child’s Child Outcomes Summary (COS) scores, or EI service quality. This limited our estimates of service need and service use; future studies are needed with information on service content and quality to confirm or refute our results. Furthermore, replication of study results should include developmental scores and COS data as they are core data elements that EI programs routinely collect as part of determining eligibility and engaging in mandated outcomes reporting. Finally, analyses were based on data that were collected at a single point in time as part of a single arm pilot trial of the YC-PEM e-PRO in EI, so causality could not be inferred. Future studies should aim for a replication longitudinally and employ a control group, which appears to be feasible, acceptable, and valuable next step [13].

## Conclusion

Electronic patient-reported outcomes (e-PRO) data may help to evaluate EI care on patient-centric outcomes for demonstrating value-based EI. This study provides evidence of the value of YC-PEM e-PRO data for demonstrating links between EI service use and participation as a patient-centric outcome, as well as yielding new knowledge about the home environment as a viable pathway to improving a young child’s participation in valued activities. Together, these results strengthen the evidence for implementing the YC-PEM e-PRO within an EI clinical workflow to further accelerate patient-centered outcomes research in EI.

## Data Availability

NIH funds through the Center for Large Data Research (CLDR) (P2CHD065702; PI: Ottenbacher) were secured to partner with the Inter-university Consortium for Political and Social Research (ICPSR) to curate the data source for public use, effective June 2019.

## Abbreviations

e-PRO: Electronic patient-reported outcome
EI: Early intervention
YC-PEM: Young Children’s Participation and Environment Measure
fPRC: Family of participation-related constructs
